# Draft Genome Sequence of *Mycobacterium* sp. Strain shizuoka-1, a Novel Mycobacterium Isolated from Groundwater of a Bathing Facility in Shizuoka, Japan

**DOI:** 10.1128/genomeA.01309-17

**Published:** 2017-11-22

**Authors:** Mitsunori Yoshida, Shinji Izumiyama, Hanako Fukano, Kanji Sugiyama, Masato Suzuki, Keigo Shibayama, Yoshihiko Hoshino

**Affiliations:** a Department of Mycobacteriology, National Institute of Infectious Diseases, Higashimurayama, Tokyo, Japan; b Department of Parasitology, National Institute of Infectious Diseases, Shinjuku, Tokyo, Japan; c Department of Research and Development, Maruma Co. Ltd, Hamamatsu, Shizuoka, Japan; d Antimicrobial Resistance Research Center, National Institute of Infectious Diseases, Higashimurayama, Tokyo, Japan; e Department of Bacteriology II, National Institute of Infectious Diseases, Musashimurayama, Tokyo, Japan

## Abstract

*Mycobacterium* sp. strain shizuoka-1 is a rapidly growing scotochromogenic mycobacterium and was isolated from well water for a bathing facility in Shizuoka Prefecture in Japan. Here, we report the draft sequence of its genome, comprising a 6.5-Mb chromosome. This mycobacterium has 83.1% identity with *Mycobacterium rhodesiae*, a human pathogen.

## GENOME ANNOUNCEMENT

Hot springs and bathing facilities are the most frequent places where *Legionella* pneumonia occurs in Japan (1). To control this fatal infection, we are collaborating with prefectural and municipal public health institutes and periodically evaluating bath water to check for contamination by *Legionella* species (1, 2). Our recent data suggest that there is a risk of legionellosis in a variety of aquatic environments, even in residential houses, not only in public baths (3). As a by-product of these examinations, we frequently isolate other varieties of mycobacteria, suggesting that they commonly live in the environmental water. This time, we isolated a novel rapidly growing scotochromogenic mycobacterium from well water for a hot bathing facility in Shizuoka prefecture, Japan. Here, we report the draft genome sequence of this mycobacterium, named *Mycobacterium* sp. strain shizuoka-1.

The strain was grown with Middlebrook 7H9 medium. DNA was extracted using the NucleoSpin plant II kit (Macherey-Nagel, Düren, Germany). The genome sequence was determined using Illumina 300 × 2 paired-end reads (4,367,175 reads) obtained with a MiSeq sequencer (Illumina, San Diego, CA, USA) (4). The reads were assembled with *Platanus* version 1.1 into 162 contigs (5). Automated annotation was carried out with the DDBJ Fast Annotation and Submission Tool (DFAST) (https://dfast.nig.ac.jp/).

The genome of *Mycobacterium* sp. shizuoka-1 is 6,533,596 bp in length, with 67.6% G+C content. The average nucleotide identity to *Mycobacterium rhodesiae* (strain DSM 44223) was 83.1%, with *M. rhodesiae* being reported as a cause of continuous ambulatory peritoneal dialysis (CAPD)-associated peritonitis (6, 7). The chromosome contains 6,207 predicted protein-coding sequences (CDS), 2 rRNAs, and 49 tRNAs. The genome sequence of *Mycobacterium* sp. shizuoka-1 represents essential data for future investigation of various environmentally contaminating mycobacteria (8).

### Accession number(s).

This whole-genome sequence has been deposited at DDBJ/ENA/GenBank under the accession numbers BEWG01000001 to BEWG01000162.
